# Difficult tracheal intubation and post-extubation airway stenosis in an 11-month-old patient with unrecognized subglottic stenosis: a case report

**DOI:** 10.1186/s40981-017-0079-4

**Published:** 2017-02-15

**Authors:** Natsuko Ohsima, Fumimasa Amaya, Shunsuke Yamakita, Yoshinobu Nakayama, Hideya Kato, Yumi Muranishi, Toshiaki Numajiri, Teiji Sawa

**Affiliations:** 1 Department of Anesthesiology, Kyoto Prefectural University of Medicine, Kajii-cho 465 Kamigyo-Ku, Kyoto, 604-0096 Japan; 2 Department of Plastic and Reconstructive Surgery, Kyoto Prefectural University of Medicine, Kajii-cho 465 Kamigyo-Ku, Kyoto, 604-0096 Japan

**Keywords:** Sevoflurane, Tracheal Intubation, Subglottic Stenosis, Difficult Tracheal Intubation, Airway Stenosis

## Abstract

**Background:**

Subglottic stenosis can lead to life-threatening difficult tracheal intubation during general anesthesia. We report a case of difficult tracheal intubation in an 11-month-old female who had unrecognized subglottic stenosis.

**Case presentation:**

The patient was scheduled for elective correction of a right accessory auricle. She was suspected of having first and second branchial arch syndrome. Preoperative physical examination was normal. Anesthesia was induced uneventfully using sevoflurane. It was not possible to pass size 4.0, 3.5, or 3.0 cuffed endotracheal tubes due to an advanced subglottic lesion. Subsequent successful intubation was achieved using a 3.0 uncuffed tube. Stridor was audible after extubation, and the patient required several days’ treatment with dexamethasone to address respiratory distress.

**Conclusions:**

We encountered unrecognized subglottic stenosis that led to difficult tracheal intubation and post-extubation airway stenosis.

## Background

Subglottic stenosis is a risk factor for difficult airway management under general anesthesia. However, this may be unrecognized in patients and only present when tracheal intubation is attempted. We report a case of unrecognized subglottic stenosis in a patient scheduled for elective surgery that led to difficult tracheal intubation and post-extubation airway stenosis that continued for several days. Written, informed consent from the patient’s parent was obtained before publication of the case report and is available on editors’ request.

## Case presentation

**Fig. 1:**
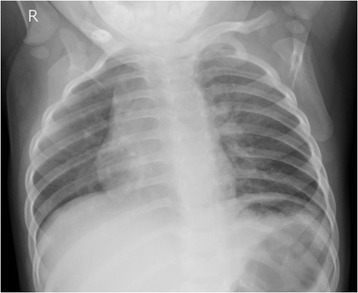
Chest X-ray images taken prior to the surgery

An 11-month-old female was scheduled for elective correction of a right accessory auricle. Her body weight was 7.1 kg and height was 69.8 cm. The patient was born at 38 weeks, with a birth weight of 2796 g and a normal Apgar score. At 4 months of age, she was suspected of having first and second branchial arch syndrome because of paralysis of the left marginal mandibular branch of the facial nerve, as well as hypotrophy of the left side of the mandible, external auditory canal, and tympanum. Preoperative investigations including laboratory tests, chest X-ray, and electrocardiograph were normal. Figure 1 demonstrates her preoperative chest X-ray. Preoperative examination of her airway and respiratory system demonstrated normal mouth opening, no apparent differences between the extremities of the mandible, and normal respiratory sounds on auscultation. We could not detect any signs predictive of difficult airway management such as a short neck, reduced immobility, or small-sized jaw.

Anesthesia was induced with inhalation of 67% nitrous oxide, 33% oxygen, and sevoflurane. Muscle paralysis was achieved by administration of 20 mg rocuronium after obtaining intravenous access. The glottic view on direct laryngoscopy using a Robertshaw blade was Cormack-Lehane Grade I. Tracheal intubation was attempted with a size 4.0 cuffed spiral tube; the size of the tube selected was based on her age. This was unable to be advanced into the subglottic region. Bag-mask ventilation was maintained during the procedure. Attempts to intubate with smaller sized tubes, up to size 3.0 cuffed, were unsuccessful. Intubation was subsequently successful using a size 3.0 uncuffed tube. No air leak around the tube was detected when airway pressures were below 30 cm H_2_O. In total, tracheal intubation was attempted four times. The first attempt was by an anesthesia resident and following attempts were by a JSA board certified anesthesiologist. The duration from induction to intubation was 28 min.

Anesthesia was maintained with inhalation of air, oxygen, and sevoflurane. The surgery proceeded uneventfully. The duration of surgery was 19 min. Residual neuromuscular blockade was reversed with 50 mg of sugammadex. The patient was extubated awake. After extubation, pulse oximetry revealed an oxygen saturation of 97–98% on room air, and stridor was not audible when she was not agitated. Fiberoptic laryngoscopic examination was performed by the otorhinolaryngologist 4 h after the extubation. Swelling or redness of the vocal cords and arytenoids could not be detected. Dexamethasone (3.3 mg) was administered intravenously.

On postoperative day (POD) 2, stridor was audible at rest, and the patient appeared fatigued. Her SpO_2_decreased from 93–95% on room air. Oxygen was commenced at 1 L/min via nasal cannula. Fiberoptic laryngoscopic examination by the same otorhinolaryngologist revealed swelling and redness of the subglottic region of the trachea. Her respiratory condition improved after treatment with 3.3 mg of intravenous dexamethasone and nebulized epinephrine.

**Fig. 2:**
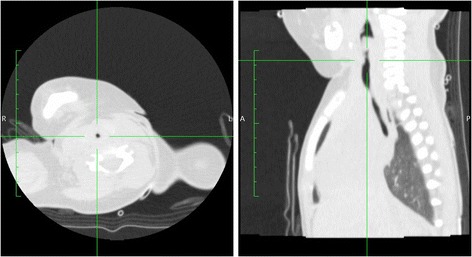
Neck CT images taken at POD 2. Subglottic stenosis of the trachea was detected. Tracheal diameter at the narrowest level was calculated as 3.2 mm. *Left*, transverse image of the neck. *Right*, sagittal image of the neck

**Fig. 3:**
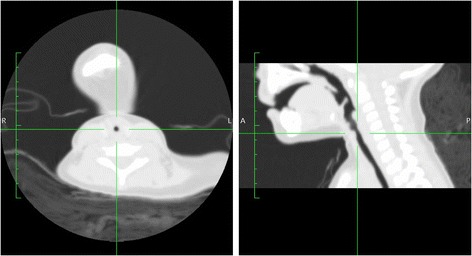
Neck CT images taken at POD 7. Tracheal stenosis was improved but still existed at the subglottic level. The sagittal plane image demonstrates the process of the anterior wall of the trachea (*arrow* in the *right panel*). *Left*, transverse image of the neck. *Right*, sagittal image of the neck

Computed tomography (CT) images of the neck showed narrowing of the subglottic portion of the trachea (Fig. 2). The diameter at the narrowest level was calculated as 2.8 mm. Daily treatment with 3.3 mg of dexamethasone was continued up to POD 4. Her respiratory condition continued to improve, and oxygen treatment was discontinued on POD 5. A CT scan of the neck done on POD 7 demonstrated that subglottic stenosis at the level of the cricoid cartilage was still present (Fig. 3), with the diameter at the narrowest level being 3.6 mm. After confirming that the patient’s respiratory condition had not worsened after discontinuing dexamethasone, the patient was discharged from the hospital on POD 7.

## Discussion

Subglottic stenosis can result in difficult tracheal intubation at induction of anesthesia [1–4]. Subglottic stenosis may be either congenital or acquired [5]. The case presented here was considered as being congenital, as the patient had not received previous tracheal intubation. Children with mild or moderate congenital subglottic stenosis have normal respiratory findings [6]. In children who underwent tracheal intubation for routine surgery, mild and moderate degrees of subglottic stenosis were found in 0.9 and 0.06% of cases, respectively [7].

Congenital subglottic stenosis is frequently associated with head and neck abnormalities [8–10]. Until now, however, there has been no report of congenital subglottic stenosis diagnosed in patients with first and second branchial arch syndrome. The histopathology of congenital subglottic stenosis includes abnormally shaped cricoid cartilage or laryngeal cleft [11]. Most frequently, the transverse diameter is shorter than normal, making the stenosis detectable on chest X-ray examination [6]. In the present case, detection of the stenosis by preoperative chest X-ray was difficult since the tracheal shape was flattened, while transverse diameter was preserved according to CT imagery. The narrowest tracheal diameter calculated, based on the CT image, was 3.6 mm on POD 7. This was much smaller than the average tracheal diameter measured at the subglottic level (for an 11-month-old, 5.0–6.5 mm in the transverse and 6.0–9.0 mm for the anteroposterior diameters) [12].

It is sometimes difficult to choose an adequately sized endotracheal tube in children. We chose an ID 4.0 mm cuffed tube for our first attempt based on the patient’s age [13]. In retrospect, this choice was slightly too large when considering recent recommendations [14]. Other predictive protocols, based on height and the fifth fingernail, are also available [15]. While we did not detect air leakage below 30 cm H_2_O with a 3.0 uncuffed endotracheal tube, we did not consider exchanging it for a smaller tube, since (1) ventilation with a tube size of 2.5 or less would be difficult, owing to increased airway resistance in this patient, (2) the planned (and actual) surgical duration was short, and (3) tube exchange in this condition may have risked inability of re-intubation and mask ventilation. However, placing a tube of this size might have led to exacerbation of congenital stenosis as in newborn cases [16]. Previous reports suggest that detection of an air leak between 10 and 30 cm H_2_O is indicative of an adequate tube size [17]. Others recommend exchanging the endotracheal tube to a smaller size if it does not leak at 40 cm H_2_O [13].

According to our literature search, no cases of unrecognized subglottic stenosis reported severe post-extubational airway stenosis with [1, 3, 4] or without [2] the prophylactic use of steroids. In contrast, in the present case, symptoms associated with airway stenosis continued for more than 24 h after surgery and necessitated restarting treatment with dexamethasone. The apparent post-extubation airway stenosis in our case could be attributed to the smaller airway size, as the patient was of a younger age than patients in previous reports.

## Conclusions

We encountered a case of undiagnosed subglottic stenosis that resulted in unexpected difficult tracheal intubation. Airway stenosis continued for 24 h after the extubation, suggesting the need for careful airway monitoring following extubation.
